# The Institute on Methods and Protocols for Advancement of Clinical Trials in ADRD (IMPACT-AD): A Novel Clinical Trials Training Program

**DOI:** 10.14283/jpad.2021.12

**Published:** 2021-04-02

**Authors:** Tyler Berkness, M.C. Carrillo, R. Sperling, R. Petersen, P. Aisen, C. Flournoy, H. Snyder, R. Raman, J.D. Grill

**Affiliations:** 1 Alzheimer's Therapeutic Research Institute, University of Southern California, San Diego, CA, USA; 2 Alzheimer's Association, Division of Medical and Scientific Relations, Chicago, IL, USA; 3 Department of Neurology, Brigham and Women's Hospital, Massachusetts General Hospital, Harvard Medical School, Boston, MA, USA; 4 Department of Radiology, Division of Nuclear Medicine and Molecular Imaging nMassachusetts General Hospital, Harvard Medical School, Boston, MA, USA; 5 Department of Neurology, Mayo Clinic College of Medicine, Rochester, MN, USA; 6 Institute of Memory Impairment and Neurological Disorders, University of California at Irvine, Irvine, CA, USA; 7 Department of Psychiatry & Human Behavior, University of California at Irvine, Irvine, CA, USA; 8 Department of Neurobiology & Behavior, University of California at Irvine, Irvine, CA, USA

**Keywords:** IMPACT-AD, training, Alzheimer's disease, ADRD, clinical Trials, diversity

## Abstract

**Background:**

Alzheimer's Disease and Related Dementias (ADRD) clinical trials require multidisciplinary expertise in medicine, biostatistics, trial design, biomarkers, ethics, and informatics.

**Objectives:**

To provide focused interactive training in ADRD clinical trials to a diverse cadre of investigators.

**Design:**

The Institute on Methods and Protocols for Advancement of Clinical Trials in ADRD (IMPACT-AD) is a novel multidisciplinary clinical trial training program funded by the National Institute on Aging and the Alzheimer's Association with two educational tracks. The Professionals track includes individuals who fill a broad variety of roles including clinicians, study coordinators, psychometricians, and other study professionals who wish to further their knowledge and advance their careers in ADRD trials. The Fellowship track includes current and future principal investigators and focuses on the design, conduct and analysis of ADRD clinical trials.

**Setting:**

The 2020 inaugural iteration of IMPACT-AD was held via Zoom.

**Participants:**

Thirty-five trainees (15 Fellowship track; 20 Professionals track) were selected from 104 applications (34% acceptance rate). Most (n=25, 71%) identified as female. Fifteen (43%) were of a non-white race; six (18%) were of Hispanic ethnicity; eight (23%) indicated they were the first person in their family to attend college.

**Measurements:**

Participants completed daily evaluations as well as pre- and post-course assessments of learning.

**Results:**

Across topic areas, >90% of trainees evaluated their change in knowledge based on the lectures as “very much” or “somewhat increased.” The mean proportion correct responses in pre- and post-course assessments increased from 55% to 75% for the Professionals track and from 54% to 78% for the Fellowship track.

**Conclusions:**

IMPACT-AD successfully launched a new training opportunity amid a global pandemic that preliminarily achieved the goals of attracting a diverse cohort and providing meaningful training. The course is funded through 2025.

## Introduction

**K**ey to the US National Plan to Address Alzheimer's Disease and Related Dementias (ADRD) will be clinical trials of therapies that are capable of slowing or preventing the onset of symptoms (1). In addition to individuals living with dementia, ADRD trials enroll participants with mild cognitive impairment and preclinical Alzheimer's disease stages, each requiring novel designs and methods (2, 3). There remains no FDA approved therapy for neuropsychiatric symptoms of ADRD (4) and these trials face unique challenges (5). ADRD trials incorporate a variety of clinical outcome measures, including cognitive, functional, and biomarker assessments (6, 7, 8). ADRD biomarkers can also be used as inclusion criteria and to support claims of disease modification (9). Across ADRD trial types, novel aspects of recruitment and retention (10), informed consent (11), and other ethical issues (12) such as the role of study partners, require sensitive attention. In short, ADRD trials are complex, multifaceted, and require unique training.

There is a dearth of qualified investigators with adequate training and expertise to conduct these complex studies (13). Such training is rarely provided through the traditional course of medical or biostatistical education. The complexity of ADRD trials requires a team science approach, often inclusive of medical doctors, neuropsychologists, biostatisticians, neuroimagers, and biomarker scientists, to name a few. The low availability of ADRD trialists, including clinical investigators, statisticians, and other experts represents a threat to the national ADRD research agenda. Not only must the pipeline of qualified trialists be increased, the makeup of this pool of investigators and research teams must be diversified (14).

A diverse team of investigators brings a multitude of ideas and perspectives to trial design and is essential to facilitate inclusive enrollment in ADRD trials (15, 16, 17, 18, 19). Diversifying study teams is a core component of the mission of the Alzheimer's Clinical Trials Consortium (ACTC). The ACTC's Inclusion, Diversity, and Education in Alzheimer's disease Clinical Trials (IDEA-CT) Committee is charged with developing goals, formulating a strategic plan, and serving as a source of oversight to support the ACTC's core values of inclusion, diversity and training in ADRD clinical trials.

To address these needs and goals, members of the ACTC IDEA-CT committee developed the Institute on Methods and Protocols for Advancement of Clinical Trials in ADRD (IMPACT-AD). IMPACT-AD is a novel multi-disciplinary clinical trial training program funded by and developed in partnership with the National Institute on Aging (NIA) and the Alzheimer's Association. IMPACT-AD is funded through 2025 with the goal of developing a network of well-trained and diverse investigators that will shape the future of the field.

In this manuscript, we describe the development of the IMPACT-AD course and the results of the inaugural iteration, which was forced to move to a virtual format due to the COVID-19 pandemic.

## Methods

### Program Structure

We designed IMPACT-AD to include two tracks of training. A “Professionals Track” focused on training ADRD clinical trials team members who sought to further their knowledge and advance their careers in ADRD trials including clinicians, study coordinators, psychometricians, and other study professionals. A “Fellowship Track” focused on training current and future principal investigators and emphasized the design, conduct, management and analysis of ADRD clinical trials.

Four committees supported the planning and conduct of IMPACT-AD. A Curriculum Committee ensured fulfillment of learning objectives. Two application review committees evaluated applicants on merit while promoting diversity in IMPACT-AD. A Program Evaluation Committee assisted in determining the short and long-term effectiveness of the course. Thirty-seven experienced clinical trial investigators, primarily composed of ACTC site PIs and unit leaders, served as course faculty (Table 1). Sixteen “core faculty” provided mentorship in protocol development to the Fellowship track trainees.

**Table 1:** Course Faculty (*Core Faculty)

Neelum T. Aggarwal, MD* Rush University	Carl V. Hill, PhD Alzheimer's Association	Michael Rafii, MD, PhD* University of Southern California
Paul Aisen, MD University of Southern California	Judith Heidebrink, MD* University of Michigan	Dorene Rentz, PhD* Harvard University Brigham and Children's Hospital
Laura Baker, PhD Wake Forest University	Gregory Jicha, MD, PhD* University of Kentucky	Robert Rissman, PhD University of Southern California University of California, San Diego
Karen Bell, MD* Columbia University	Gustavo Jimenez-Maggiora, MBA University of Southern California	Laurie Ryan, PhD National Institute on Aging
Jeffrey Burns, MD* Kansas University	Jason Karlawish, MD University of Pennsylvania	Stephen Salloway, MD* Brown University Butler Hospital
Maria Carrillo, PhD Alzheimer's Association	David Knopman, MD Mayo Clinic, Rochester	Mary Sano, PhD Mount Sinai Hospital
Michael Donohue, PhD* University of Southern California	Holly Lynch Fernandez, JD University of Pennsylvania	Gopalan Sethuraman, PhD* University of Southern California
Hiroko Dodge, PhD* Oregon Health Sciences University	Kristina McLinden, PhD National Institute on Aging	Amanda Smith, MD* University of Southern Florida
Mark Espeland, PhD* Wake Forest University	Bri McWhorter Activate to Captivate	Heather Snyder, PhD Alzheimer's Association
Howard Fillit, MD Alzheimer's Drug Discovery Foundation	John Olichney, MD* University of California, Davis	Reisa Sperling, MD Harvard University Brigham and Children's Hospital
Daniel Gillen, PhD* University of California, Irvine	Ronald Petersen, MD, PhD Mayo Clinic, Rochester	David Sultzer, MD University of California, Irvine
David Geldmacher, MD* University of Alabama, Birmingham	Jeremy Pizzola University of Southern California	Christopher Van Dyck, MD Yale University

### Outreach and Application Process

We employed a breadth of strategies to ensure our goal of a robust and diverse course applicant pool. A Request for Applications (RFA) announced the course and outlined the application requirements, including: 1) personal statement; 2) letter of support from a mentor or supervisor; and 3) NIH biosketch. For the Fellowship track, a draft protocol using the ACTC Protocol Synopsis template was also required. The RFA was disseminated widely. The Alzheimer's Association's International Society to Advance Alzheimer's Research and Treatment (ISTAART) shared the RFA with their mailing list (n=2100) and active research awardees (n=540), including their diversity fellowship recipients. The NIA distributed the RFA to 2019 grantees (n=2300) and to alumni of the Butler-Williams Scholars Program. We sent the RFA to the ACTC steering committee members and investigative teams for numerous studies coordinated by the USC Alzheimer's Therapeutic Research Institute (n=530) and to the National Alzheimer's Coordinating Center's mailing list (n=780). Applications were submitted through the Alzheimer's Association's centralized ProposalCentral web-based grant management service.

### Selection Criteria

Each application was reviewed and scored by no fewer than five reviewers including the course co-directors. Selection criteria included: 1) demonstration of passion and commitment for ADRD clinical trials and likelihood of future involvement in ADRD research; 2) level of support from a supervising faculty member; 3) publication record; and for the Fellowship track 4) the quality of the draft protocol. Two remote study sections were convened to discuss applications and select the class of 2020.

### Course Curriculum

The course curriculum included didactic lectures and active learning workshops over four days. Professionals track trainees participated for two days; Fellowship track trainees participated for the duration of the course. Didactic lectures addressed fundamental concepts in clinical trials as well as unique aspects within ADRD (Table 2). Three active learning workshops addressed scientific communication, trial publications, and securing funding. For the Fellowship track, additional protocol workgroups focused on trial design and protocol development skills. Workgroups were comprised of three Fellowship track trainees and at least three course core faculty members, including two clinical and one biostatistical faculty. Protocol workgroups focused on five specific topics: 1) trial designs; 2) selecting a sample and developing inclusion criteria; 3) selecting a primary (and other) outcome measures; 4) statistical analysis plans; 5) safety monitoring and other conduct considerations.

**Table 2:** Didactic Lectures and Workshop Content

Both Tracks	Fellowship Track Only	Protocol Workgroups
ADRD Trials and Objectives	Traditional vs. Adaptive Design Choices	Introductions and Discussion of Trial Designs
Participant-Related Issues	ADRD Trial Populations, Indications, and Outcomes	Choosing a Sample; Developing Inclusion/Exclusion Criteria
ADRD Trial Ethics	Working with a Statistician Collaborator	Selecting a Primary (and other) Outcome Measure
Design Features in ADRD Trials	Study Management: Serving as Principal Investigator	Statistical Analysis Plans
Critical Evaluation of Literature*	Securing Trial Funding*	Safety Monitoring and Other Conduct Considerations
Presentation and Communication Skills*	Reflections from a Study Session Panel*	


* Workshops

### Course Evaluations

We collected evaluations on all sessions and lectures within each session. Trainees assessed several aspects of the course including the value of each covered topic, prior knowledge of the topic and the effect on the participant's knowledge of the lecture. Trainees scored sessions using Likert response scales tailored to each question (e.g., “Very strong”, “Strong”, “Moderate” and “Weak” as options for “What was your prior knowledge of this topic?”).

We used pre- and post-course evaluations of knowledge to determine the overall educational value of the course. Separate post-test evaluations were performed at the conclusion of Days 2 (end of the Professionals track) and 4 (end of the Fellowship track). We compared the group scores pre- and post-course completion.

## Results

### Characteristics of Applicants and Selected Trainees

We received 104 eligible applications including 48 for the Fellowship track and 56 for the Professionals track. Sixteen individuals applied to both tracks. Most applicants were female and nearly half identified as being from a non-white race and/or Hispanic/Latino ethnicity (Table 3). Twenty-three applicants (22%) indicated that they were the first in their family to attend college. Forty-six (44%) were from Institutions outside of the ACTC network.

**Table 3:** IMPACT-AD Applicant and Trainee Demographics

Characteristic (Self-Reported)	Professionals Track		Fellowship Track	
	Applied	Selected	Applied	Selected
Female Sex	39 (69.6%)	14 (70%)	28 (58.3%)	11 (73.3%)
Race				
African American or Black	8 (14.3%)	3 (15%)	8 (16.7%)	4 (26.7%)
Asian	7 (12.5%)	1 (5%)	9 (18.8%)	3 (20%)
White or Caucasian	38 (67.9%)	13 (65%)	27 (56.3%)	7 (46.7%)
Multi-Racial	2 (3.6%)	2 (10%)	3 (6.3%)	1 (6.7%)
Other	1 (1.8%)	1 (5%)	1 (2.1%)	0 (0%)
Hispanic/Latino Ethnicity	4 (7.3%)	2 (10.5%)	6 (12.8%)	4 (28.6%)
First in Family to Attend College	16 (28.6%)	4 (20%)	7 (14.6%)	4 (26.7%)
ACTC Institution	32 (57.1%)	12 (60%)	26 (54.2%)	10 (66.7%)

Thirty-five trainees (15 in the Fellowship track and 20 in the Professionals track) were selected to participate in the course, resulting in a 34% acceptance rate. Among selected trainees, the majority were female. Seven (20%) identified as African American or Black, four (11%) as Asian, twenty (57%) as White/Caucasian, three (8.5%) as multi-racial, one (3%) as Other race and six (18%) identified as being of Hispanic ethnicity. Eight trainees (23%) identified as being the first person in their family to attend college. Eleven (31%) held Professional degrees (e.g. MD, DDS, MBBS), fifteen (43%) held Doctorate degrees (e.g. PhD, PsyD), six (17%) held Master's degrees, and three (9%) had a Bachelor's degree. Thirteen (37%) were from institutions outside of the ACTC network. For the Fellowship track, eleven (73%) trainees proposed trials of nonpharmacological interventions, and four (27%) proposed drug trials.

### Course Evaluations and Assessment of Learning

Each day of the course achieved at least an 80% response rate for program evaluations. Table 4 overviews the course evaluations for each of the sessions. On average, lecture topics were rated as “essential” by 76% and “valuable” by 22% of trainees. None of the topics received any assessment of “not necessary.”

**Table 4:** Evaluation Summaries

	Session Value			Previous Knowledge				Knowledge After Lecture			
	Essential (%)	Valuable (%)	Not Necessary (%)	Very Strong (%)	Strong (%)	Moderate (%)	Weak (%)	Very Much Increased (%)	Somewhat Increased (%)	Slight Increase (%)	No Change (%)
AD Trial Design	64.7	30.2	0	25.2	26.0	38.6	10.3	50.7	41.9	7.4	0
Participant-Related Issues	75.9	20.7	0	20.7	40.2	33.3	5.7	49.4	39.1	6.9	4.6
ADRD Trial Ethics	86.9	13.1	0	35.7	45.2	17.9	1.2	55.9	39.3	4.8	0
Statistical Design & Analysis	75	23.2	0	21.4	14.3	50.9	13.4	39.3	53.6	7.1	0
Populations, Indications and Outcomes	79.8	20.2	0	13.5	24.1	52.9	9.6	59.6	36.6	2.9	1.0
Statistical Considerations	82.4	17.6	0	8.3	8.3	61.1	22.2	52.8	36.1	5.5	5.5
Study Management	68.3	30.0	0	26.7	25	41.7	6.7	53.3	23.3	15	8.3
Total	76.2	22.1	0	21.6	26.2	42.3	9.9	51.6	38.5	7.1	2.8


Mean scores are presented for each session, which included 2–6 lectures of varying lengths.

Across lecture topics, 22%, 26%, 42%, and 10% of trainees rated their prior knowledge of topics as “very strong,” “strong,” “moderate,” and “weak”, respectively. The areas deemed as the greatest need by trainees (most responses of weak prior knowledge) included those in statistical design and analysis, with 22% of trainees identifying their prior knowledge as weak.

**Figure 1:**
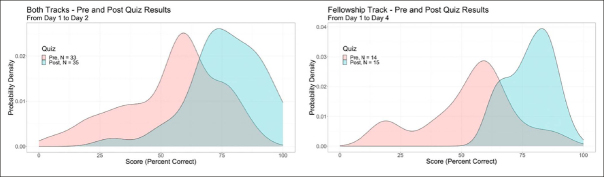
Pre- and Post-Course Quizzes of Course Learning

Across topic areas, 52%, 39%, 7%, and 3% of trainees self-reported their change in knowledge based on the lectures as “very much increased,” “somewhat increased,” “slightly increased,” and “no change”, respectively. Based on pre- and post-course assessments, each track demonstrated a positive effect of the course on trial knowledge (Figure 1). The mean proportion correct responses for the Professionals track increased from 55% to 75%. The Fellowship track improved from 54% to 78% correct responses.

Mean performance on pre- and post-course assessments of knowledge are presented for days 1 vs. 2 (panel A), which included both the Professionals and Fellowship tracks (n=33 pre and n=35 post), and for days 1 vs. 4 (panel B), which included only the Fellowship track (n=14 pre and n=15 post).

## Discussion

IMPACT-AD was envisioned as an annual in-person course held at the ACTC Coordinating Center/University of Southern California's Alzheimer's Therapeutic Research Institute in San Diego, CA. The COVID-19 pandemic caused by the novel coronavirus SARS-CoV-2 forced implementation of a virtual format for the inaugural iteration of IMPACT-AD. As a result, we significantly adjusted the course's structure and format in an effort to accommodate trainees' time zones and ensure achievement of the course objectives. The course days had to be shortened and morning and evening activities were cancelled. The planned educational content remained largely intact. The results presented here indicate that these efforts were successful.

The course achieved its primary educational goals. Trainees received instruction in key topics related to ADRD interventional research and showed increased knowledge as a result of their training. Long-term evaluations will assess whether trainees continue their roles in ADRD trials, whether they achieve career advances supported by their participation in the course, and whether Fellowship track trainees successfully conduct their proposed trials.

Increasing investigator diversity is an important goal for the ACTC and specifically the IDEA-CT committee and more broadly for the field of ADRD research (20, 21). The inaugural IMPACT-AD course achieved the goal of including a diverse cohort of trainees. Trainees were diverse in sex, race and ethnicity, as well as professional backgrounds and current positions. Notably, eight trainees were the first in their families to attend college. While these diverse trainees were generally already working in ADRD trials, the course aims to give them added tools to be successful, advance in their careers, and inspire them to continue their work in the field.

A main goal of the IMPACT-AD course is to establish a network of peers that can remain connected, learn from each other, and support each other's careers. Establishing this sense of camaraderie was made more challenging by the necessitated virtual conduct of the course. In partnership with the trainees, however, we created an IMPACT-AD Alumni Platform through the professional networking site LinkedIn. Thirty-one of 35 trainees (89%) have enlisted in this group. The Alumni Platform plans to interact virtually to discuss recent publications, plan and hold seminars, and discuss available funding and collaboration opportunities. The group is led by an IMPACT-AD Alumni Committee, composed of four trainees (two from each track). We also plan to hold an in-person event with the Class of 2020 at the earliest safe opportunity and will pursue other opportunities to connect alumni from subsequent iterations of the course.

IMPACT-AD has received funding to hold an annual course for the next four years. Based on the first year's conduct, several changes are planned. Applicants will be required to select only one track. We anticipate holding informational webinars to answer potential applicant questions and offer guidance on the qualities that distinguished successful applications. Course content will be reorganized, emphasizing fundamental information on trial design (randomization, blinding, etc.) earlier in the agenda. We also anticipate developing some recorded lectures or webinars that will be offered to participants prior to the course to address the areas acknowledged by trainees as greatest needs (i.e., basic design and statistical analysis). We aim to improve evaluation completion rates.

## Conclusions

The first year of the IMPACT-AD course was successful, despite unforeseen challenges resulting from the COVID-19 global pandemic. A diverse cohort of trainees was recruited and trained, and available data suggest that the training was effective. With strong partnerships with the NIA, the Alzheimer's Association, and ACTC, the IMPACT-AD course is poised to continue its mission to train and diversify the next generation of ADRD trial investigators.

*Funding:* This work was supported by NIA U13AG067696, NIA U24AG057437, and Alzheimer's Association SG-20-693744. JDG is supported by NIA AG066519 and NCATS UL1 TR001414.

*Ethical standards:* This paper does not describe human subjects research and therefore there was no IRB approval needed.
